# Development of a Wireless and Passive SAW-Based Chemical Sensor for Organophosphorous Compound Detection

**DOI:** 10.3390/s151229793

**Published:** 2015-12-03

**Authors:** Fang-Qian Xu, Wen Wang, Xu-Feng Xue, Hao-Liang Hu, Xin-Lu Liu, Yong Pan

**Affiliations:** 1 Zhejiang University of Media and Communications, Hangzhou 310018, China; xufangqian2006@163.com; 2 State Key Laboratory of Acoustics, Institute of Acoustics, Chinese Academy of Sciences, No.21, North 4th Ring West Road, Beijing 100190, China; xuexufeng@mail.ioa.ac.cn (X.-F.X.); huhaoliang11@mails.ucas.ac.cn (H.-L.H.); liuxinlu1987@foxmail.com (X.-L.L.); 3 State Key Laboratory of NBC Protection for Civilian, Yangfang, Changping District, Beijing 102205, China; panyong71@sina.com.cn

**Keywords:** chemical sensor, organophosphorous compounds, fluoroalcoholpolysiloxane (SXFA), surface acoustic wave (SAW), wireless and passive

## Abstract

A new wireless and passive surface acoustic wave (SAW)-based chemical sensor for organophosphorous compound (OC) detection is presented. A 434 MHz reflective delay line configuration composed by single phase unidirectional transducers (SPUDTs) and three shorted reflectors was fabricated on YZ LiNbO_3_ piezoelectric substrate as the sensor element. A thin fluoroalcoholpolysiloxane (SXFA) film acted as the sensitive interface deposited onto the SAW propagation path between the second and last reflectors of the SAW device. The first reflector was used for the temperature compensation utilizing the difference method. The adsorption between the SXFA and OC molecules modulates the SAW propagation, especially for the time delay of the SAW, hence, the phase shifts of the reflection peaks from the corresponding reflectors can be used to characterize the target OC. Prior to the sensor fabrication, the coupling of modes (COM) and perturbation theory were utilized to predict the SAW device performance and the gas adsorption. Referring to a frequency-modulated continuous wave (FMCW)-based reader unit, the developed SAW chemical sensor was wirelessly characterized in gas exposure experiments for dimethylmethylphosphonate (DMMP) detection. Sensor performance parameters such as phase sensitivity, repeatability, linearity, and temperature compensation were evaluated experimentally.

## 1. Introduction

Obviously, chemical sensors with high sensitivity and fast response for organophosphorous compound (OC) detection in real time are necessary for responding to the threats of terrorism and environmental pollution. Among a wide range of available chemical sensors, surface acoustic wave (SAW)-based chemical sensors exhibit many unique advantages such as high sensitivity, ambient-temperature operation, fast response, low cost, easy reproducibility, and good stability [1,2,3,4,5,6]. Typical SAW-based chemical sensor systems are composed of a differential SAW oscillator array and a chemical interface coated onto the SAW propagation path of the sensing SAW device. The SAW propagation is modulated as the vapor is adsorbed selectively and reversibly on the chemical interface. The corresponding shift in SAW velocity induces changes in the differential oscillation frequency, which are used to characterize the target vapor. Many SAW chemical sensor prototypes have been reported, and even commercialized in OC sensing [7]. In addition to the above characteristics and working configuration, the SAW sensor exhibits another unique advantage, which is the option of implementing wireless and passive measurements for gas sensing. Recently, a variety of passive and wireless sensors for many physical quantities like temperature, pressure, torque, strain, and even moisture were realized using SAW technology [8,9,10,11,12]. In particular, Wang *et al*. proposed some passive SAW-based sensors for wirelessly sensing some toxic or harmful gases like CO_2_, NO_2_, and some other volatile organic compounds, where the phase signal from the reflectors of the SAW reflective delay line configuration induced by the gas adsorption between the sensitive interface and target gas was picked for gas sensing. Clear sensor responses were observed in wireless measurements [13,14,15,16]. Lieberzeit *et al*. reported some considerations on wireless and passive SAW sensors utilizing RFID tags for mass-sensitive detection of humidity and vapors, with a detection limit down to 50 ppm for tetrachloroethene and 1% for relative humidity [17]. Huang *et al*. developed a passive wireless hydrogen SAW sensor utilizing Pt-coated ZnO nanorods [18]. Westafer *et al*. presented the characterization of a dispersive SAW reflective delay line for sensing ozone in air at ppb concentration [19]. The above works provide a good starting point for development of a real practical wireless and passive SAW chemical sensor.

**Figure 1:**
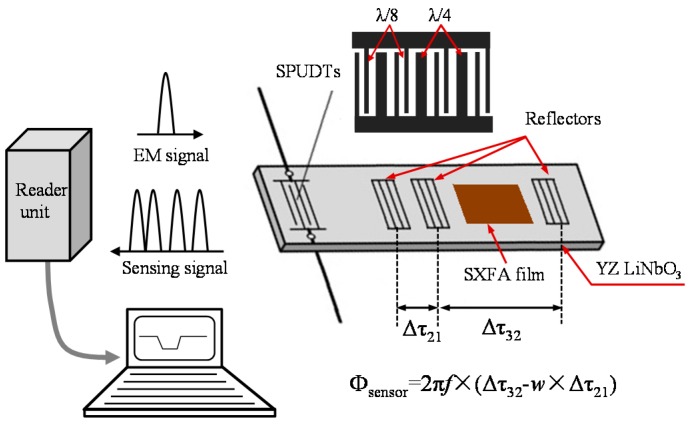
Schematic and working principle of the proposed SAW chemical sensor.

In this paper, a wireless and passive SAW chemical sensor system was developed for OC sensing referring to a frequency-modulated continuous wave (FMCW)-based reader unit. The schematic and working principle of the sensor are illustrated in Figure 1. The proposed sensor system is composed of a SXFA-coated SAW reflective delay line, FMCW-based reader unit, and the connected antennas. The use of fluoroalcoholpolysiloxane (SXFA) was frequently reported for chemical interfaces for OC detection because of its low crystallinity and glass transition temperature, and high permeability [20,21]. The SAW reflective delay line acted as the sensor chip, in which single phase unidirectional transducers (SPUDTs) and three shorted grating reflectors were placed in a row along the SAW propagation direction on an YZ LiNbO_3_ piezoelectric substrate. SXFA film was coated onto the surface between the second and third reflectors of the SAW device for DMMP sensing. The first reflector of the SAW device was used for temperature compensation utilizing the difference method. When the SPUDTs receive electromagnetic (EM) energy from the reader unit through the connected antenna, the SAW is generated by the SPUDTs through the piezoelectric effect, and propagates towards the reflectors. The propagating SAW is reflected partially from the reflectors, and reconverted into EM waves by the SPUDTs and transmitted to the reader unit. The adsorbed OCs in the SXFA film modulate the SAW propagation, resulting in shifts in SAW velocity and attenuation, and changes in time delay of the reflection peaks from the reflectors. Therefore, target OC concentrations can be extracted by evaluating the differential phase signals, Φ_sensor_, as Φ_sensor_ = 2 × π × *f* × (Δτ_32_ − w × Δτ_21_), where *f* is the operation sensor frequency, Δτ_32_ and Δτ_21_ are the measured changes in time delays among the three reflection peaks from the peaks including the information of environmental temperature and target species concentration, and *w* is the ratio of the second-to-third reflector distance to the first-to-second reflector distance [13]. It is obvious that the proposed chemical sensor presents many advantages:

1. The sensor chip is maintenance-free because it is passive, and does not require any power supply to operate;
2. The working environment can be very hazardous such as contaminated and high voltage areas because of the wireless measurement;
3. Temperature compensation can be accomplished well by using the sensor chip design through the differential method [13,14,15].

Prior to the sensor fabrication, the coupling of modes (COM) and perturbation theory were utilized to predict the SAW device performance and describe the gas adsorption of the sensor. Then, the developed SAW chemical sensor system was characterized wirelessly in DMMP gas experiments. The phase sensitivity, repeatability, and temperature compensation were investigated experimentally.

## 2. Design Considerations

### 2.1. Reader Unit

**Figure 2:**
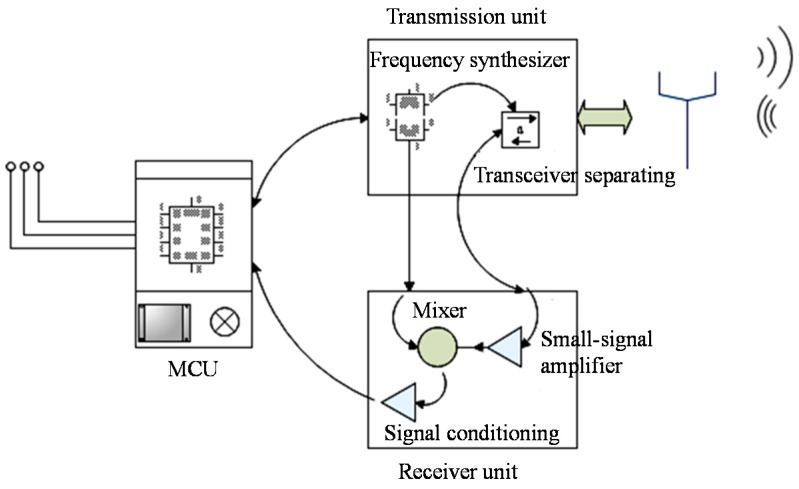
The structure of the used FSCW reader unit.

Impulse radar interrogation units are usually applied for wireless sensor systems because of their simple structure. However, due to the required fast sampling and switching circuitry, they are costly. Therefore, the frequency stepped continuous wave (FSCW) radar technique, which generates a continuously variable frequency sine wave excitation signal was utilized in this study [22]. Using impulse signals in the frequency domain instead of those in the time domain, the speed requirements of the AD chip were greatly reduced, and as a result, the cost of the sensor system decreases. A schematic of the FSCW-based reader unit is presented in Figure 2. The main control unit (MCU) consists of a single chip microprocessor (SCM: C8051F065) and its auxiliary circuitry. The signal transmission unit was composed of frequency synthesizer and transceiver separating parts. The frequency synthesizer primarily produces sine wave signals with different frequency for the local oscillator (LO) port of the transmission and mixer circuit. The transceiver separating part is targeted to separate the high-frequency transmission signal and the reception signal, making them flow forward following the predetermined signal without interfering or conflicting with each other. The signal receiver unit was mainly composed of a small-signal amplifier, mixer and signal conditioning circuits. The small-signal amplifier circuit is set to amplify the received signal, and prevent noise from drowning the signal. The mixer circuit is used for mixing the received signal and the LO signal, modulating the received signal to a low frequency range which can be sampled. The signal rectifying circuit is responsible for amplifying and rectifying the low frequency signal from the mixer, and ensuring that the signal conforms to the requirements of the follow-up AD sample circuit.

The module diagrams of the corresponding PC modeling software include the T/F conversion, reflection peak extraction, phase determination, and model building. Among the four sectors of the PC modeling software, the time/frequency (T/F) conversion part mainly works for converting signals in the time domain to frequency domain by using the fast Fourier transform (FFT). Before FFT, some preprocessing steps such as zero-padding and windowing to the time domain data should be done. The reflection peak extraction section is mainly responsible for extracting the peak position in the frequency domain of the three reflected signal peaks from the reflectors of the SAW device. As to the distortion in phase determination caused by the phase exceeding 360°, the phase model reported in [23] is utilized, which eliminates the distortion of phase and increasing the accuracy of detection by setting three reflectors to obtain the differential time-delay. Combining the detection results of the phase detection module, the phases of the three reflected signals could be acquired and determined, respectively, and sent to the model building part, which is designed to determine the curve of the relation between phase shift and temperature and gas adsorption [24].

### 2.2. Design on the SAW Sensor Chip

A SAW reflective delay line configuration was used as the sensor chip in the proposed chemical sensor system. High signal-to-noise (S/N), low insertion loss, sharp reflection peaks in the time domain, and few spurious noises are required for sensor performance improvement [23]. Hence, an YZ LiNbO_3_ piezoelectric crystal was chosen as the substrate owing to its large electromechanical coupling factor K^2^ (4.5%), which allows strong reflectivity for the reflectors and lower insertion loss. Single phase unidirectional transducers (SPUDTs), which direct most of the SAW energy to the direction of the reflectors were used to structure the SAW device. Hence, improved S/N and lower insertion loss were achieved. Shorted grating reflectors were adopted owing to their strong reflectivity [25].

The operation frequency of the sensor is set to 434 MHz, to also meet the requirement of the sweep frequency bandwidth (less than 30 MHz) of the reader unit, and to obtain sharp reflection peaks in the time domain, the finger pairs in the SPUDTs was designed as 15, that means the transducer has 15 periods. As mentioned in Figure 1, three shorted grating reflectors were placed in the SAW propagation path, whereby the third reflector was used for gas sensing where the SXFA film was coated onto the surface between the second and third reflector, and the first one was used for temperature compensation. Additionally, to keep adequate separation between environmental noise echoes and reflection peaks, the time interval between the SPUDTs and the first reflector was designed to larger than 1.2 μs. Also, to achieve equal amplitude for all the reflection peaks in the time domain, the reflector lengths were appropriately controlled, that is, the farther away from the SPUDTs, the longer the reflector is. To eliminate the phase distortion, the difference in time delay between the first and second reflector is designed to 0.15 μs, and the ratio of second-to-third reflector distance to first-to-second reflector distance is set to 13. Details of the design parameters of the SAW device are listed in Table 1.

**Table 1:** Design parameters for the SAW device.

Design Parameters	Value
Operation frequency (MHz)	434
Piezoelectric substrate	YZ LiNbO_3_
Thickness of the Al electrodes (nm)	300
Distance between SPUDT and the first reflector (λ: wavelength)	250.25
Distance between the second and the third reflector (λ)	399.75
The length of the second reflector(λ)	2.25
Acoustic velocity (m/s)	3492
Aperture (λ)	80
Number of SPUDT finger pairs	15
Distance between the first and the second reflector (λ)	30.75
Length of the first reflector (λ)	2.25
Length of the third reflector (λ)	4.25

To predict the device performance, the typical coupling of modes (COM) theory, which provides a very efficient way of optimizing SAW devices [25], was utilized. For simulation of the SAW reflective delay line configuration composed by SPUDTs and three shorted grating reflectors, the COM model was utilized to analyze the SPUDTs and reflectors, respectively. By using the extracted mixed P-matrix of the SPUDTs, reflectors, and the gaps among the SPUDTs and reflectors, the admittance matrix of whole device Y ($Y=\left[\begin{array}{cc} y_{11} & y_{12}\\ y_{21} & y_{22} \end{array}\right]$) can be deduced, and then, the reflection coefficient S_11_ of the SAW device can be obtained by:

(1) $$S_{11}=\frac{(Y_{G}-y_{11})\times (Y_{G}+y_{22})+y_{12}\times y_{21}}{(Y_{G}+y_{11})\times (Y_{G}+y_{22})-y_{12}\times y_{21}}$$

where Y_G_ is the resource and load inductance. 

**Figure 3:**
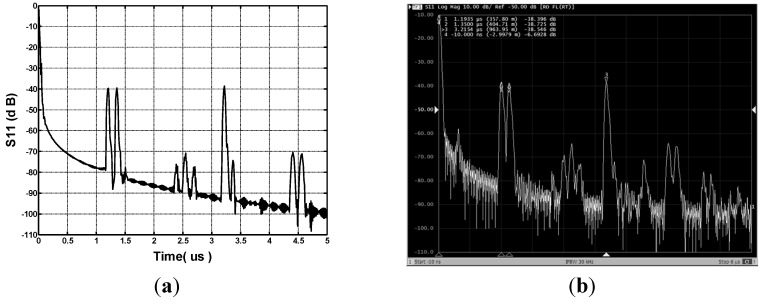
Simulated (**a**) and measured (**b**) S_11_ in time domain of the SAW device.

Using the parameters listed in Table 1 and the FFT program, the SAW device used for gas sensing was simulated to obtain the S_11_ in the time domain. Figure 3a shows the simulated S_11_ in time domain in the case of YZ LiNbO_3_, aluminum SPUDTs with 15 finger pairs and 100λ aperture size, and three shorted grating reflectors. Three reflection peaks from the three reflectors are observed clearly in the time domain.

### 2.3. Theoretical Analysis of the Sensor Response

As described in Figure 1, the adsorption between the SXFA and DMMP modulates SAW propagation. As a polymer material, SXFA offers excellent selectivity, lower detection limits, and ambient-temperature operation, however, the viscoelastic nature of the SXFA will induce nonlinearity in the sensor response and additional acoustic attenuations in the process of SXFA deposition and gas adsorption, so prior to sensor development, it is essential to predict the sensor response of the SXFA-coated chemical sensor. Actually, perturbation theory was frequently used to describe the polymer-coated SAW chemical sensor, which provide a good way to optimize the sensor configuration [26]. The perturbation on SAW propagation from the sensitive interface deposition can be analyzed by:

(2) $$\begin{array}{l} \Delta \gamma /k_{0}=\Delta \alpha /k_{0}-j\Delta v/v_{0}=\sum_{i=1}^{3}\frac{c_{i}\beta_{i}M_{i}}{\omega }\tanh(j\beta_{i}h)\\ \beta_{i}=\omega \sqrt{\frac{\rho -E^{i}/{v_{0}}^{2}}{M^{i}}}\\ E^{1}=\frac{4G(3K+G)}{3K+G},E^{2}=G,E^{3}=0\\ M^{1}=M^{2}=G,M^{3}=K \end{array}$$

where *k*_0_ and *v*_0_ denote the unperturbed wave number and SAW velocity, respectively. Δ*γ*/*k*_0_ represents the fractional perturbation in the complex wave propagation factor, Δ*α*/*k*_0_ notes the change in attenuation per wave number, while Δ*v*/*v*_0_ is the fractional change in propagation velocity. The terms *c* and *ρ* are the SAW-film coupling parameter and the density of polymer material, and *h* is the polymer thickness. *G* and *K* denote the shear horizontal (SH) modulus and the bulk modulus, which specify the viscoelastic properties of the polymer. Usually they are both complex numbers, and their real parts represent the storage moduli, whereas the imaginary parts mean the loss moduli.

Then, the density *ρ* and thickness *h* of the polymer sensitive interface will vary with the concentration of the adsorbed species C:

(3) $$\begin{array}{l} \rho \left(C\right)\;=\rho_{0}+\kappa C_{v}/\left(1+\kappa C_{v}/\rho_{v}\right)\\ h\left(C\right)\;=h_{0}\left(1+\kappa C_{v}/\rho_{v}\right) \end{array}$$

where *C_v_* is the gas concentration, and *κ* is the partition coefficient that was well defined by Grate *et al.* [27]. *ρ_0_* and *ρ_v_* are the densities of the polymer material and target gases.

**Figure 4:**
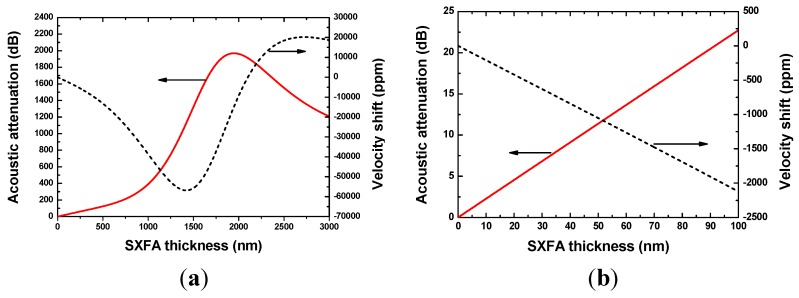
The effect on SAW propagation of the SXFA deposition at (**a**) thicker SXFA and (**b**) thin SXFA film.

**Figure 5:**
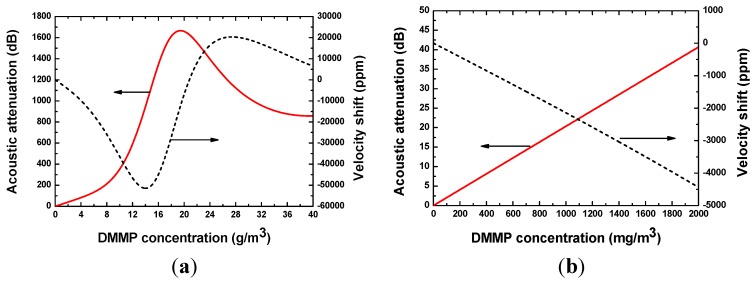
The effect on SAW propagation in the gas adsorption at (**a**) high and (**b**) low DMMP concentration.

SXFA is a glassy-rubbery polymer material with density of 1.447 g/cm^3^, and its shear modulus G and bulk modulus K are 9.3 × 10^9^ Pa + 1.2 × 10^9^ Pa and 10 × 10^9^ Pa + 10 × 10^9^ Pa, respectively [20]. Dimethylmethylphosphonate (DMMP) with density of 1.145 g/cm^3^ and molecular weight of 124 was assumed as the target gas. The DMMP concentration is assumed to 0~1000 mg/m^3^ in the simulation. The partition coefficient logκ of SXFA towards to DMMP is around 6.4 [20]. As for wireless sensing, the operation sensor frequency is set to 434 MHz. Hence, the effect on SAW propagation from the SXFA deposition can be simulated using Equations (2) and (3), as shown in Figure 4. The induced acoustic attenuation in the SXFA deposition process was computed by using the expression Δ*α*/*k*_0_ × L/0.0183 [20], where L is the SXFA coated acoustic wave propagation path length (~350λ). In Figure 4, the existed resonation phenomenon in velocity shift and loss change demonstrates the viscoelastic characteristics of the SXFA. It is well known that larger acoustic attenuation will seriously affect the sensor performance. To reduce the acoustic attenuation (less than 10 dB), SXFA thickness was advised to be less than 50 nm. Additionally, under the given SXFA thickness of 50 nm, the relationship among the acoustic attenuation, velocity shift and adsorbed DMMP concentration was calculated as shown in Figure 5. Like Figure 4, the viscoelastic nature of the SXFA itself also leads to a obvious resonance phenomenon, that is, nonlinear velocity shifts and larger acoustic wave attenuation will occur at higher gas concentrations. Obviously, in trace gas concentrations of less than 200 mg/m^3^, the induced acoustic wave attenuation is low enough to be neglected, and the shift in velocity is linear, that is a mean linear sensor response will be expected.

## 3. Technique Realization

### 3.1. SAW Sensor

Using the design parameters mentioned in Table 1, the SAW reflective delay line configuration was reproducibly fabricated on a YZ LiNbO_3_ piezoelectric substrate as the sensor element as shown in Figure 1. Three hundred nm Al-strips were deposited onto the YZ-LiNbO_3_ substrate utilizing standard photolithographic techniques. After the device dicing-sawing, the SAW devices were characterized by using a network analyzer, as shown in Figure 3b.Three reflection peaks were clearly observed from the reflectors in the time domain, which shows high S/N ratio, sharp peaks, and few spurious signals. The first reflection peak appears at 1.1935 μs, and the corresponding amplitude of S_11_ is 38.4 dB. The second and the last reflection peaks occurred at 1.35 μs and 3.215 μs with S_11_ amplitudes of 38.7 dB and 38.54 dB, respectively. Good uniformity was observed in all of the amplitudes of the reflection peaks. Also, the measured results agree well with the COM simulation.

**Figure 6:**
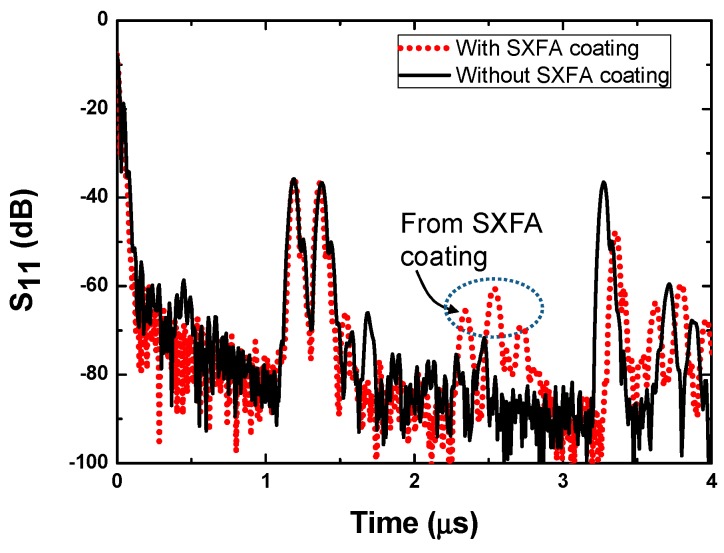
The measured S_11_ of the SAW device before and after SXFA coating.

In our work, SXFA was chosen as the sensitive interface for DMMP detection, which was coated onto the LiNbO_3_ substrate surface of the developed SAW reflective delay line by means of solvent-evaporation. Here, the SXFA was synthesized by reaction of polyallylmethylsiloxane andhexafluoro-acetone (HFA). The solvent-evaporating method was applied for the SXFA coating owing to its simple operation and low cost. Usually, the effectiveness of the solvent evaporating method to produce a stable polymer coating is significantly determined by the solvent type. In this study, toluene was utilized as the solvent. Before the SXFA coating, the YZ LiNbO_3_ surface was cleaned of any contaminants by a routine cleaning procedure involving rinsing in piranha solution (V(H_2_SO_4_):V(H_2_O_2_) = 3:1), a DI water rinse and drying by N_2_. Then, a 0.1 μL solution of 0.8 g/L SXFA/toluene was deposited on the cleaned piezoelectric substrate surface between the second and third reflectors three consecutive times at different place along the SAW propagation path between the second and third reflector. The deposited SXFA was characterized by using the atomic force microscopy (AFM) which approximately demonstrated the surface roughness, and its thickness is measured as ~41 nm. It is clear that there are some obvious changes in time delay and amplitude of S_11_ of the third reflection peaks before and after SXFA deposition, as depicted in Figure 6. Because of the mass loading effect of the SXFA coating, the velocity along the SAW propagation path between the second and third reflector decreases, leading to an increase in delay time. Also, the viscoelastic nature of the SXFA induces acoustic wave attenuation, resulting in a decrease in the amplitude of the third reflection peak.

**Figure 7:**
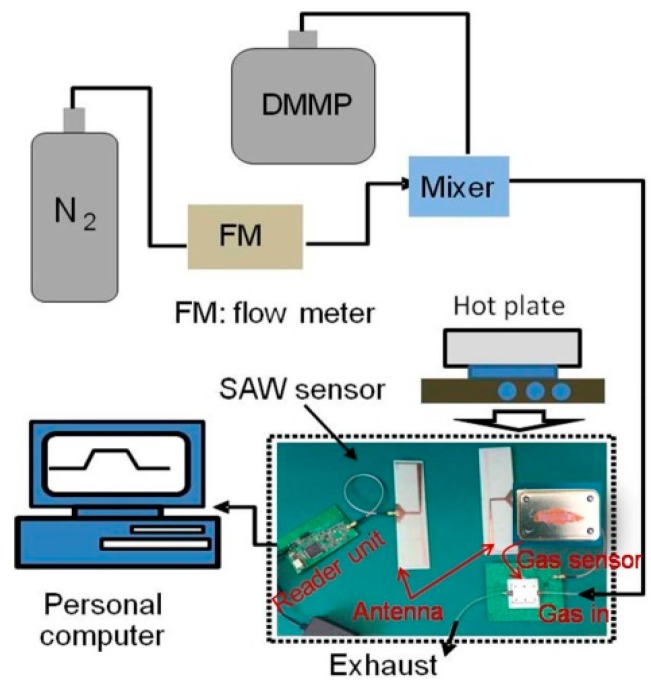
The experimental set up for characterizing the wireless SAW sensor.

Also, For wireless measurement, two 2-dimensional planar antennas (18 cm × 5 cm) operating at 434 MHz central frequency and 30 MHz bandwidth were made from a 0.4 mm thick FR4 substrate (dielectric constant k = 4.2) with Cu electrodes and electrically connected to the SAW sensor chip and reader unit. Based on the design considerations described in Section 2.1, the 434 MHz FSCW-based reader unit was fabricated, as shown in Figure 7.

### 3.2. Sensor Experimental Setup

Then, the developed SXFA coated SAW sensor chip was placed in a surface nickel-plated 500 mL aluminum gas chamber with two separate gas channels, and connected to the antenna.The completed wireless SAW chemical sensor system was composed of the FSCW-based reader unit, SXFA-coated sensor, and connected antennas. The experimental setup shown in Figure 7 consists of the wireless sensor system, dual channel gas flow controller system, hot plate, and thermometer. The SAW sensor was exposed to N_2_ and DMMP gas alternately via the gas path. The sensor signals were acquired by the reader unit and processed by the self-compiled software in the personal computer in real time.

## 4. Wireless Measurement of the SAW Sensor

### 4.1. DMMP Adsorption

Before gas sensing experiments, the basic phase noise of the sensor system, which is from the Gaussian noise in the wireless measurement, was measured at ~20 cm request distance, ~±2° of phase noise was observed 120 s.

**Figure 8:**
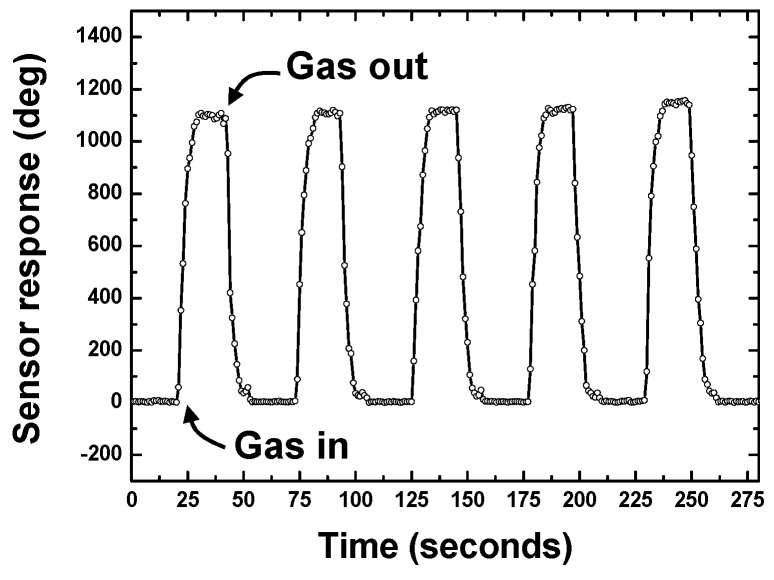
The repeatability measurement of the sensor system.

Then, the developed SXFA-coated wireless and passive sensor was used for DMMP sensing to investigate its repeatability. The reflection peak S_11_ of the sensor chip was picked by the reader unit at ~20 cm request distance. The adsorbed DMMP molecules in SXFA film induced time deviations of the resultant reflection peaks, and the corresponding phase signal was picked up and recorded to evaluate the DMMP concentration because the phase signal provides much higher resolution over the time shift of the reflection peak. Figure 8 shows a typical phase response profile obtained from five consecutive 50 s on-off exposures to 50 mg/m^3^ DMMP in pure N_2_ at room temperature (25°C) and a gas flow speed of 1 L/min. Here, the phase response was recordedevery1 s so that one point on the graph corresponds to a 1-sinterval.The sensor response plotted in Figure 8 denoted a rapid response rise on exposure to DMMP that reaches approximately 80% of the equilibrium (saturation) value in 7 s. When the gas was removed by the N_2_, the phase response returned to ~70% of its initial baseline value within 6 s. Relatively fast rise and fall times were observed. Also, it is obvious that the developed sensor shows excellent repeatability of the response to DMMP.

### 4.2. Temperature Effect on Sensor Response

**Figure 9:**
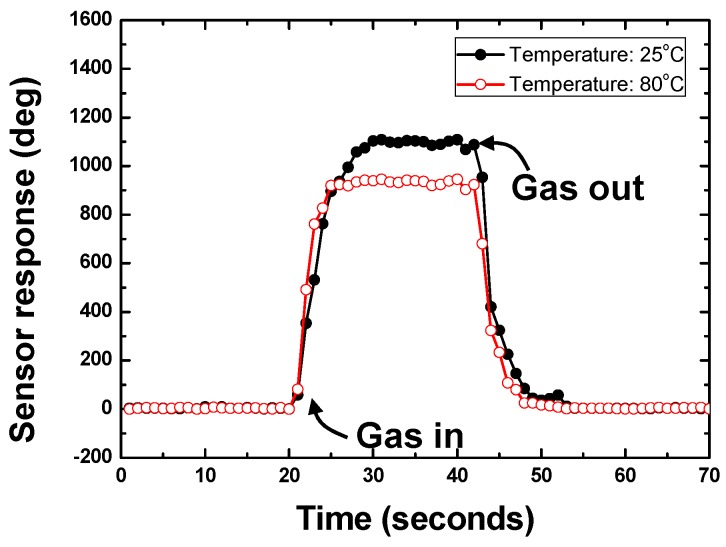
Temperature effect analysis on sensor response.

It is well known that the environmental temperature influences significantly the sensor performance by causing shifts in the baselines and altering the adsorption interactions between the interface and the target gases. To study the temperature effect on the sensor response, the testing environment temperature was varied from room temperature to 80 °C by the hot plate. The measured phase responses towards 50 mg/m^3^ under different testing temperature are summarized in Figure 9.

Even though the differential method was utilized, there are still obvious deviations in the sensor responses at different testing temperature. The differential method can effectively reduce the temperature effect from the thermal expansion of the piezoelectric substrate, but it is helpless for the temperature effect from the thermal expansion of the SXFA film and gas adsorption activities at elevated temperature. A promising approach to solve such issue would be to add a reference material to eliminate the temperature influence from the SXFA film itself using the differential structure. As the premise, the physical properties of the reference materials should be similar to the SXFA. This work will be the focus in our further investigation.

### 4.3. Sensitivity Evaluation

**Figure 10:**
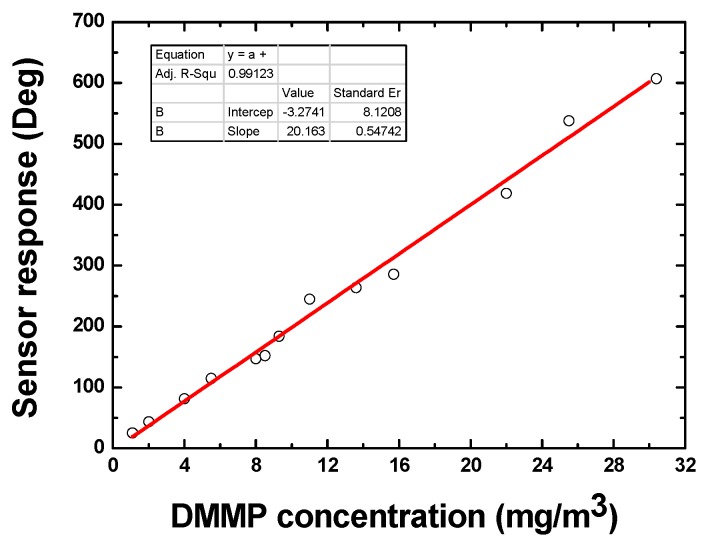
The sensitivity evaluation of the developed SAW sensor.

The developed SAW sensor was exposed to various concentrations of DMMP at 25 °C to evaluate its sensitivity. The DMMP concentration was increased from 1 mg/m^3^ to 30 mg/m^3^. Figure 10 describes the obtained phase response of the sensor depending on various DMMP concentrations. It is obvious that as the concentration increased, the sensor phase signal also increased with approximate linearity. The sensitivity in DMMP concentration range of 1~30 mg/m^3^ was evaluated as 20.1°/(mg/m^3^) with linearity of 0.99123. Considering the basic noise of ±2° and phase signal of ~20° response to 1 mg/m^3^ DMMP, the detection limit of the sensor is estimated at less than 0.5 mg/m^3^.

## 5. Conclusions

A new wireless and passive SAW-based chemical sensor composed of a FSCW based reader unit, a SXFA-coated reflective delay lines, and two planar antennas connected to the reader unit and SAW device was proposed for OC sensing. The coupling of modes (COM) model and perturbation theory were used to predict the device performance and gas adsorption at the sensitive interface. The response of the developed SAW sensor towards DMMP was characterized in gas experiments. Excellent linearity and repeatability were achieved. The sensitivity and detection limit are evaluated as 20.1°/(mg/m^3^) and 0.5 mg/m^3^, respectively.
